# Primary testicular lymphoma with subcutaneous masses as the sole manifestation of the first relapse and central nervous system lymphoma as the second relapse: A case report and literature review

**DOI:** 10.3892/ol.2014.2052

**Published:** 2014-04-09

**Authors:** ZUNGUO DU, YIN WANG, PING ZHU, HAIYAN LENG, FENG TANG, XIAOPING XU, ZI CHEN

**Affiliations:** 1 Department of Pathology, Huashan Hospital, Fudan University, Shanghai 200040, P.R. China; 2 Department of Neuropathology, Huashan Hospital, Fudan University, Shanghai 200040, P.R. China; 3 Department of Hematology, Huashan Hospital, Fudan University, Shanghai 200040, P.R. China

**Keywords:** primary testicular lymphoma, subcutaneous soft tissue, relapse

## Abstract

Primary testicular lymphoma (PTL) accounts for ~1% of all non-Hodgkin’s lymphomas and has a marked tendency for systemic relapse. The current study presents a unique case of testicular diffuse large B-cell lymphoma of non-germinal center B-cell subtype, with subcutaneous masses as the sole manifestation of the first relapse and central nervous system lymphoma as the second relapse. Subcutaneous relapse and subsequent brain relapse are extremely rare signs of PTL dissemination. The patient received methotrexate-based combined chemotherapy and achieved a partial response. This case presents a rare pattern of treatment failure in this malignant clinical entity.

## Introduction

Primary testicular lymphoma (PTL) is a rare disease accounting for ~1% of all non-Hodgkin’s lymphomas (NHLs) and 5% of all testicular malignant tumors (1–4). PTL has a marked tendency to involve additional extranodal sites, including the central nervous system (CNS), contralateral testicles and Waldeyer’s ring, however, the CNS is the most common site of involvement. Due to its rarity, the treatment of PTL has not been standardized. Despite adequate treatment, the majority of PTL patients relapse within the first two years of treatment and therefore, the prognosis of PTL is poor.

The current study presents an extremely rare case of PTL presenting initially as stage I extranodal (IE) disease with the successive recurrence of the subcutaneous soft tissue and CNS lymphoma. Patient provided written informed consent.

## Case report

In September 2004, a 65-year-old male with no significant past medical history presented to the Huashan Hospital (Shanghai, China) with a painless testicular swelling. Physical examination of the patient revealed a right testicular mass and a system-by-system review of the body functions was unremarkable, including the absence of B symptoms. In addition, the Eastern Cooperative Oncology Group performance status was 0. The patient underwent a right orchiectomy, and pathological examination revealed a diffuse large B-cell lymphoma (DLBCL) of non-germinal center B-cell (GCB) subtype. The results of the patient’s immunohistochemical analysis were as follows: Leukocyte common antigen^+^, cluster of differentiation (CD)20^+^, CD10^−^, B-cell lymphoma (BCL)6^−^, multiple myeloma oncogene-1^+^, BCL2^+^, CD3^−^, CD56^−^, S100^−^, epithelial membrane antigen^−^, CD30^−^ and anaplastic lymphoma kinase^−^, with a Ki67 score of 80%. A further staging work-up included a bone marrow biopsy with negative observations. Computed tomography (CT) scans and ultrasound examinations also showed no evidence of enlarged lymph nodes or extranodal involvement. Analysis of the cerebrospinal fluid (CSF) was normal and the serology for human immunodeficiency virus (HIV) was negative. The clinical Ann Arbor stage was determined as stage I extranodal (IE) with an age-adjusted International Prognostic Index score of 0.

The patient completed four cycles of cyclophosphamide/doxorubicin/vincristine/prednisone (CHOP) chemotherapy between September 2004 and December 2004. During the treatment and follow-up, CT scans and ultrasound examinations showed no evidence of lymphoma progression.

**Figure 1:**
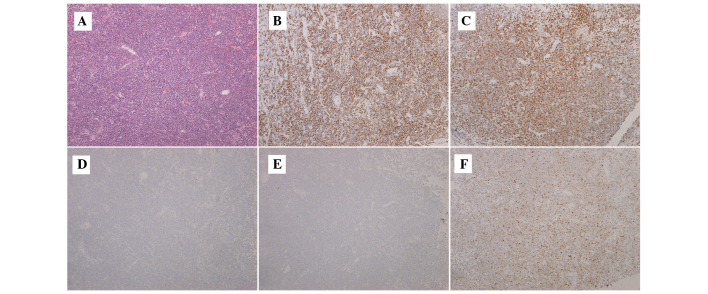
Pathological examination of a subcutaneous mass. (A) Hematoxylin and eosin staining (magnification, ×100). (B) CD20^+^, (C) BCL2^+^, (D) CD10^−^, (E) BCL6^−^ and (F) MUM1^+^ immunostaining (magnification, ×100). CD, cluster of differentiation; BCL, B-cell lymphoma; MUM1, multiple myeloma oncogene-1.

In July 2012, the patient visited the clinic due to the appearance of three subcutaneous masses above the right knee. The physical examination was unremarkable, with the exception of three mobile and rock-hard subcutaneous masses, which were ~3–4 cm in diameter. The masses were not swollen or painful and the local temperature was within the normal range. A biopsy was performed on the new lesions and the pathological observations revealed a DLBCL of non-GCB subtype, with identical morphological and immunohistochemical features to those of the original testicular DLBCL of non-GCB subtype (Fig. 1). A whole body PET-CT was subsequently performed and showed no other sites of lymphoma involvement. The patient declined chemotherapy, but underwent 20 fractions of regional irradiation with a total dose of 40 cGy and consequently experienced resolution of the right leg masses. The post-treatment CT scan and ultrasound examination showed no additional evidence of lymphoma.

In January 2013, the patient was admitted to the Department of Neurosurgery due to progressive weakness in the right extremities. A neurological examination upon admission showed right-sided hemiparesis and brain magnetic resonance imaging revealed a lesion in the left frontal lobe. In addition, the CSF and ocular slit lamp examinations were normal and the serology for HIV was negative. A biopsy of this lesion was performed and the histopathological and immunohistochemical examinations revealed DLBCL of non-GCB subtype. The patient was then transferred to the Department of Hematology and the CT scans of the chest, abdomen and pelvis, and the ultrasound examination of the lymph nodes and left testicle were normal. The bone marrow biopsy and aspiration showed no involvement. The patient subsequently received two cycles of methotrexate-based combined chemotherapy (2 g/m^2^ methotrexate intravenously on day one, 100 g/m^2^ teniposide intravenously on days two and three, and 15 mg dexamthasone on days one to three; for one cycle of 28 days). Brain magenetic resonance imaging showed that the lesion had evidently contracted and the patient remained symptom-free. Subsequently, the patient refused chemotherapy. The patient was closely followed for three months, but was then lost to follow-up.

## Discussion

The current study presents a rare case of PTL with subcutaneous masses as the sole manifestation of first relapse and CNS lymphoma as the sole manifestation of second relapse.

PTL is a rare entity, with a reported annual incidence of ~0.26 per 100,000 individuals in Denmark (5). However, it is also the most common testicular neoplasm in males over 50 years old (6). One retrospective study that was reported by the International Extranodal Lymphoma Study Group (IELSG) included 10 countries and 373 patients with primary testicular DLBCL and found that the median age at diagnosis was 66 years. Testicular DLBCL is characterized by a particularly high risk of extranodal relapse even in cases with localized disease at diagnosis. In this study, the median follow-up was 7.6 years and 195 patients (52%) relapsed; CNS relapse was detected in 56 patients (15%). In addition, the median overall survival (OS) time was 4.8 years (7). In a population-based study utilizing the Surveillance, Epidemiology and End Results database to identify primary testicular DLBCL patients diagnosed between 1980 and 2005, 769 patients were identified and the median age at diagnosis was 68.0 years. The incidence of DLBCL was found to increase over time and the median OS time was 4.6 years. The study confirmed a short overall survival time and a higher risk of continuous relapse of primary testicular DLBCL (4).

In total, 68–77.8% of PTL cases are DLBCL (8,9) and gene expression profiling can further subdivide DLBCL into GCB and activated B-cell [ABC (non-GCB)] subtypes, which have different clinical outcomes. In addition, the ABC subtype has a more aggressive clinical behavior than the GCB subtype (10–12). Previously, two studies have shown that almost all testicular DLBCLs belong to the non-GCB subtype. Al-Abbadi *et al* (13) studied 18 cases of testicular DLBCL and found that 89% of them belonged to the non-GCB subtype and all exhibited high proliferative activity. Furthermore, Li *et al* (14) also showed that 16 out of 17 testicular DLBCL cases belonged to the ABC subtype. However, two other studies have shown contrasting results. Kemmerling *et al* (15) presented the immunohistochemical analysis of 18 cases of PTL and found that 15 cases were classified as DLBCL, showing no significant prevalence of the ABC subtype (9/15, 60%) compared with the GCB subtype (6/15, 40%). The survival analysis showed that patients with GCB subtype DLBCL exhibited a trend towards a longer OS time than the patients with ABC subtype DLBCL, however, no statistically significant difference was observed. In addition, Hasselblom *et al* (16) reported that 29 testicular DLBCL patients could be classified into nine (31%) cases of GCB phenotype and 20 (69%) cases of non-GCB phenotype, however, no difference was identified in the event-free survival or OS time between the two groups. The statistical results may be affected by the small sample size and the different single center studies. Therefore, PTL cases from multiple institutes must be enrolled and analyzed in future studies.

PTL is an immune-privileged site-associated lymphoma, and in order to escape immunological surveillance, the lymphoma cells must develop an immune escape phenotype (17,18). A common aberration leading to immune escape is the loss of human leukocyte antigen expression, while an additional common aberration is a high level of somatic hypermutation. Primary central nervous system lymphoma (PCNSL) is also an immune-privileged site-associated lymphoma and may exhibit the same immune escape ability as PTL. However, these two types of rare lymphoma have significantly different presentations. PTL has a marked tendency to involve additional extranodal sites, including other immune-privileged sites. The CNS is the most commonly involved site. However, the relapse of PCNSL is almost (90–95%) confined to the CNS and few studies have reported the testis involvement of PCNSL. These phenomena indicate that PTL is much more aggressive than PCNSL and has unique mechanisms of invasion, but the exact mechanism of this aggressive behavior remains unknown. The aberrant expression of adhesion molecules may be an important factor, as the adhesion molecules mediate the cell-cell and cell-matrix interactions, affecting the homing and migration of lymphoma cells, which are important in metastatic processes. One previous study (19) detected the expression of integrins and other adhesion molecules in the testicular lymphoma cells and matrix. A few adhesion molecules, including CD49f/very late activation antigen (VLA)-6, CD49d/VLA-4, CD54 and CD62L, were detectable in a small number of lymphomas, however, the expression of other adhesion molecules was lacking. This expression pattern was indicative of high metastatic potential. Furthermore, Kawakami *et al* (20) reported that testicular lymphoma tissues showed hypermethylation of the tumor-suppressor genes, including E-cadherin, Ras association (RalGDS/AF-6) domain family member 1 and retinoic acid receptor β, which have been implicated in the pathogenesis of human cancer. The study partially explained the mechanism of the dysexpression of adhesion molecules. Dysadherin is a recently described cell membrane glycoprotein, which exhibits an anti-cell-cell adhesion function and downregulates E-cadherin. A study that detected the expression of E-cadherin and dysadherin in eight primary testicular B-cell lymphomas by immunohistochemistry identified that dysadherin was highly expressed in all cases and found to correlate with aberrant E-cadherin expression (21).

Due to the rarity of PTL, the optimal strategy for treatment remains unclear, however, an almost universal agreement on the treatment of PTL has been reached. According to the different stages of the disease, the treatment varies; for the early stage (stages I/II), the standard treatment has not yet been established, but orchiectomy is required. Post-orchiectomy systemic treatment decreases the risk of relapse, and CHOP and CHOP-like regimens are the mainstay of chemotherapy. One previous study of 373 cases of testicular DLBCL showed that anthracycline-based chemotherapy, CNS prophylaxis and contralateral testicular irradiation may improve the outcome of PTL (7). As CNS relapse continues to be a major problem for PTL patients, routine CNS prophylaxis is recommended by the majority of physicians, however, the best strategy remains debatable; radiation therapy and intrathecal chemotherapy may be favorable. A retrospective study by Mazloom *et al* (22) showed that scrotal radiation therapy is associated with an improved OS. Furthermore, the addition of the anti-CD20 monoclonal antibody, rituximab, to the chemotherapy has led to a marked improvement in the treatment of B-cell lymphoma. The study also showed that rituximab CHOP-based chemotherapy as intrathecal chemotherapy may improve OS. However, the population-based study did not achieve the anticipated improvement in the survival of testicular DLBCL patients in the rituximab era, although, it does not imply that rituximab can not add value to the treatment of PTL. An additional study also showed that patients with testicular DLBCL exhibited significantly worse survival times in the rituximab era (23). Further studies are therefore required. For advanced disease, patients must be treated according to the guidelines for the treatment of advanced-stage DLBCL. The standard therapeutic option for patients with stage III/IV disease is conventional anthracycline-containing combined chemotherapy, however, chemotherapy plus rituximab with the addition of prophylactic scrotal radiotherapy and intrathecal chemotherapy is the most positive option. The study by the IELSG showed an apparently improved outcome in advanced-stage DLBCL patients following anthracycline-based chemotherapy, CNS prophylaxis and contralateral testicular irradiation (7). The optimal therapy for patients with relapsed PTL has not yet been defined in prospective trials, however, the therapeutic strategy should be similar to other relapsed NHLs. In addition, the treatment should be decided according to age, performance status, organ function and previous treatments.

PTL has a predilection for spreading to unusual extranodal sites, including the CNS, contralateral testicles and Waldeyer’s ring. However, the localized subcutaneous relapse and subsequent brain relapse observed in the present case is an extremely rare sign of PTL dissemination.
